# Effects of an evidence service on community-based AIDS service organizations' use of research evidence: A protocol for a randomized controlled trial

**DOI:** 10.1186/1748-5908-6-52

**Published:** 2011-05-27

**Authors:** Michael G Wilson, John N Lavis, Jeremy M Grimshaw, R Brian Haynes, Tsegaye Bekele, Sean B Rourke

**Affiliations:** 1 McMaster Health Forum, Hamilton, Canada; 2 Centre for Health Economics and Policy Analysis, McMaster University, Hamilton, Canada; 3 Ontario HIV Treatment Network, Toronto, Ontario, Canada; 4 Department of Clinical Epidemiology and Biostatistics, McMaster University, Hamilton, Canada; 5 Department of Political Science, McMaster University, Hamilton, Canada; 6 Clinical Epidemiology Program, Ottawa Hospital Research Institute, Ottawa, Canada; 7 Department of Medicine, University of Ottawa, Ottawa, Canada; 8 Institute of Population Health, University of Ottawa, Ottawa, Canada; 9 Health Information Research Unit, McMaster University, Hamilton, Canada; 10 Centre for Research on Inner City Health, St. Michael's Hospital, Toronto, Canada; 11 Department of Psychiatry, University of Toronto, Toronto, Canada

## Abstract

**Background:**

To support the use of research evidence by community-based organizations (CBOs) we have developed 'Synthesized HIV/AIDS Research Evidence' (SHARE), which is an evidence service for those working in the HIV sector. SHARE consists of several components: an online searchable database of HIV-relevant systematic reviews (retrievable based on a taxonomy of topics related to HIV/AIDS and open text search); periodic email updates; access to user-friendly summaries; and peer relevance assessments. Our objective is to evaluate whether this 'full serve' evidence service increases the use of research evidence by CBOs as compared to a 'self-serve' evidence service.

**Methods/design:**

We will conduct a two-arm randomized controlled trial (RCT), along with a follow-up qualitative process study to explore the findings in greater depth. All CBOs affiliated with Canadian AIDS Society (n = 120) will be invited to participate and will be randomized to receive either the 'full-serve' version of SHARE or the 'self-serve' version (a listing of relevant systematic reviews with links to records on PubMed and worksheets that help CBOs find and use research evidence) using a simple randomized design. All management and staff from each organization will be provided access to the version of SHARE that their organization is allocated to. The trial duration will be 10 months (two-month baseline period, six-month intervention period, and two month crossover period), the primary outcome measure will be the mean number of logins/month/organization (averaged across the number of users from each organization) between baseline and the end of the intervention period. The secondary outcome will be intention to use research evidence as measured by a survey administered to one key decision maker from each organization. For the qualitative study, one key organizational decision maker from 15 organizations in each trial arm (n = 30) will be purposively sampled. One-on-one semi-structured interviews will be conducted by telephone on their views about and their experiences with the evidence service they received, how helpful it was in their work, why it was helpful (or not helpful), what aspects were most and least helpful and why, and recommendations for next steps.

**Discussion:**

To our knowledge, this will be the first RCT to evaluate the effects of an evidence service specifically designed to support CBOs in finding and using research evidence.

**Trial registration:**

ClinicalTrials.gov: NCT01257724

## Background

Community-based organizations (CBOs) are important stakeholders in health systems [1,2] because they provide a wide spectrum of programs and services to the members of their community, link with other health and social services to help provide care, and advocate for broader system-level supports. As with other health system stakeholders (*e.g.*, healthcare providers and health system managers and policymakers) it is important for CBOs to use research evidence to inform their programs, services and advocacy. To do this, they need support in finding and using research evidence to help them plan and deliver more effective and cost-effective programs and strengthen health systems.

However, there are many potential challenges related to research use. Barriers that have been consistently identified across different sectors include the complexity of research evidence, organizational barriers, lack of available time, poor access to current literature, lack of timely research, lack of experience and skills for critical appraisal, unsupportive culture for research, lack of actionable messages in research reports, and limited resources for implementation [3-7]. Given these barriers, it is not surprising that, generally, a lack of uptake of research evidence has been noted in many different sectors [8-12].

While there are strategies for supporting the use of research evidence by clinicians [13,14], and health system managers and policymakers [15-20], there is still an important gap in the availability of specific strategies for CBOs [21]. Many existing strategies for supporting the use of research evidence are based on experience and anecdotal evidence rather than on rigorous evidence of effects [15,22,23]. Moreover, strategies designed for supporting the use of research evidence by healthcare organizations and governments may not be relevant to the specific contexts and capacity of CBOs. To begin to fill this gap, we have developed an evidence service which for those working in the HIV sector, which is entitled 'Synthesized HIV/AIDS Research Evidence' (SHARE -- see below for a detailed description).

Efforts to facilitate the use of research evidence often focus on four clusters of knowledge translation activities ('producer push,' facilitating 'user pull,' 'user pull,' and 'exchange' efforts) [24], and the SHARE database primarily fits within two of these strategies. First, SHARE constitutes an effort to facilitate 'user pull' by allowing users to easily identify relevant synthesized research evidence and access user-friendly summaries when they identify the need for it. In addition, SHARE also constitutes a 'producer push' effort by providing periodic email updates that highlight synthesized research evidence that has been newly added to the database. This type of activity largely promotes awareness of newly synthesized research evidence, but it could also have more direct impact on the use of synthesized research evidence by profiling systematic reviews that address issues that CBOs may be grappling with at a particular time. What SHARE does not include are 'user pull' mechanisms (*i.e.*, target audiences incorporating prompts for research evidence in their decision-making processes and developing their capacity to find and use research evidence) or 'exchange' efforts, which focus on the producers and users of researchers building partnerships and working collaboratively in the production and interpretation of research evidence [24].

## Objectives

Our objective is to evaluate whether, how, and why this 'full serve' evidence service increases the use research evidence by key decision makers in CBOs as compared to a 'self-serve' evidence service.

## Methods/design

We will conduct this trial using a sequential explanatory mixed methods design [25], beginning with the two-arm randomized controlled trial (RCT), and then following up with a qualitative process study to explore the RCT findings in greater depth. The trial will run for 10 months, which includes a two-month baseline period where all participants receive the 'self-serve' evidence service, a six-month period where the intervention group will receive the 'full-serve' evidence service and the control group will continue to receive the 'self-serve' evidence service, and a final two-month period where both groups will receive the 'full-serve' version of SHARE.

## RCT methods and design

### Study population and recruitment

Community-based HIV/AIDS organizations in Canada provide a number of programs and services to people living with or affected by HIV, which may include prevention initiatives, individual or group counseling/support, and community outreach and/or education. In addition, organizations in Canada are situated in diverse geographic settings ranging from dense urban settings to rural, northern, and/or remote settings, with some focused on specific at-risk populations and/or cultural or ethnic groups.

We will draw our sample from those organizations affiliated with the Canadian AIDS Society and from relevant provincial HIV/AIDS networks (*e.g.*, the Ontario AIDS Network), and send an organizational invitation to the executive director and management team (if applicable). The invitation will indicate that if they are interested in having their organization participate, access to SHARE will be provided to all interested staff. Given that SHARE is currently only provided in English, we will exclude organizations that do not have at least one key decision maker who is comfortable participating and corresponding in English.

To ensure clarity in our study recruitment, we will outline that consent from the executive director is required for the organization to participate. We will also indicate that we require one key organizational decision maker to fill out a brief survey measuring their intention to use research evidence (see the Outcomes section for more detail on the survey) on behalf of their organization at baseline and again at the completion of the trial. We will request that the executive director complete the survey, but will indicate that they can delegate to another manager provided the manager has a decision-making role about programs, services, and advocacy, and provided the manager does not include the conduct of research among their core responsibilities. Because the overall intent of the intervention is to support the use of research evidence in decisions about CBOs' programs, services, and advocacy, we deemed it most appropriate for the executive director (or another manager) to complete the survey because they would have the most impact on whether research evidence is used to inform decisions.

Based on the membership list provided by the Canadian AIDS Society on their website, there are 120 CBOs available to draw the sample from. Drawing on previous experience with this sector, we expect to achieve an approximate response rate of 70%. To increase our response rate, the Canadian AIDS Society will send out an email to all its members, encouraging them to participate by highlighting the importance of the trial. We will provide additional incentive to enroll in the trial by holding a draw where we will select three organizations to receive prizes (gift cards) worth $500, $250 and $100.

### Interventions

We will run a two-arm RCT with a 'full-serve' evidence service (SHARE) as the intervention arm and a 'self-serve' version as the control arm. The components of each version of SHARE are outlined in Table 1 and described below.

**Table 1:** Components of the 'full-serve' and 'self-serve' evidence service

Evidence service components	'Full-serve' SHARE	'Self-serve' Control
1. Access to records for HIV-relevant systematic reviews*	X	X

2. Searchable database - Reviews retrievable using taxonomy of topics related to HIV/AIDS and open text search	X	

3. Email updates highlighting newly added reviews	X	

4. Access to user-friendly summaries produced by us or by others	X	

5. Links to scientific abstracts	X	X*

6. Peer-relevance assessments†	X	

7. Links to full-text (when publicly available)	X	

8. Access to worksheets that help CBOs find and use research evidence	X	X

* The 'self-serve' version will be provided as a listing of reviews grouped by year of publication with titles hyperlinked to their scientific abstract.

†Based a 5-point scale that asks how useful the reviews is and through a user-forum provided for each review record

### Intervention arm: 'full serve' evidence service

Organizations allocated to this study arm will receive access to a 'full-serve' version of SHARE, which provides:

1. an online searchable database of HIV-relevant systematic reviews (retrievable based on a taxonomy of topics related to HIV/AIDS and open text search - see Additional file 1: Appendix 1 for the taxonomy of topics);

2. periodic email updates (at least one per month), which will profile the types of new reviews recently added to the database (*e.g.*, the number of Cochrane reviews) and provide a brief overview of the range of topics addressed by the new reviews;

3. access to user-friendly summaries produced by us or by others (when available);

4. links to scientific abstracts;

5. peer relevance assessments, which involves periodic requests (contained in the single record for each review) to complete a brief assessment of how useful the information in the newly added review is (one question with a five-point scale - see Additional file 2: Appendix 2 for additional details) with the average score posted once an assessment is completed;

6. an interface for participants to leave comments (up to 250 characters in length) in the records of systematic reviews in the database (*e.g.*, if a participant wants to leave a comment indicating the review was useful and why);

7. links to full-text articles (when publicly available); and

8. access to worksheets that help CBOs find and use research evidence

To provide access to user-friendly summaries (see component three above) we will provide links to user-friendly summaries produced by nine groups (when available) from around the world: Australasian Cochrane Centre (AAC) Policy Liaison Initiative, Database of Abstracts of Review of Effects (DARE), Effective Healthcare Research Programme Consortium, Evidence AID, Health Knowledge Network, Health-Evidence.ca, Reproductive Health Library, Rx for Change, and Supporting Policy Relevant Reviews and Trials (SUPPORT)[18,26-34].

### Control arm

Organizations allocated to the control group will only be provided website access to a listing of systematic reviews that are organized by year of publication with links to the record on PubMed (or another publicly available source when not available on PubMed) and access to worksheets that help CBOs find and use research evidence.

### Randomization

After consenting to participate in the trial, we will use simple randomization to assign organizations to receive either the 'full-serve' or the 'self-serve' evidence service. The list of participating organizations will be sent to a statistician (TB) who will assign a unique ID number to each organization, conduct the randomization, and keep both the key linking the organizations to their ID and the randomization log in a secure password protected folder at the Ontario HIV Treatment Network to provide a clear audit trail. We will perform simple randomization sampling using the SAS SELECTSURVEY procedure to assign equal numbers of organizations to the 'full-serve' and the 'self-serve' groups. The procedure will be performed with a fixed seed so that the sampling can be replicated if needed. The statistician will then provide the list of unique IDs with the results of the randomization to the SHARE database administrator at the Ontario HIV Treatment Network (external to the research team) who will provide individuals from each participating organization with access to the 'full-serve' or 'self-serve' versions of SHARE. This will require the SHARE database administrator to have access to the key linking the unique IDs to the organizations but it will remain concealed from the research team.

Prior to the start of the trial, all organizations will be requested to provide a list of emails of management and staff interested in receiving access to SHARE, which will be provided to the SHARE database administrator at the Ontario HIV Treatment Network. We will then send bi-monthly emails to the executive director (or another delegated staff member for correspondence) to identify any staff that have either joined or left the organization in order to accurately track usage at the organizational level. The SHARE database administrator at the Ontario HIV Treatment Network will send the updates to individuals affiliated with organizations with access to the 'full-serve' version of SHARE (the updates will be written by MGW and checked by the co-investigators). The statistician (TB) is a member of the study team but will only be involved with randomization at the start of the trial and the data analysis upon completion of the trial. Therefore, participants and all investigators except the statistician (TB) and the SHARE database administrator will be blinded to group assignment.

### Outcomes

Measuring the impact of knowledge transfer and exchange (KTE) interventions, such as the evidence service proposed here, poses significant challenges as there is a long chain of factors between a KTE intervention such as SHARE and the health status of clients of CBOs or of broader populations [10,35]. For example, it has been demonstrated that assessing the impact of KTE interventions on the practice of physicians poses challenges due to the fact that many factors other than the practice guidelines or recommendations that were disseminated may influence how practices are changed [36-38].

Given these constraints, our primary and secondary outcomes for the trial are proxy measures for research use. The primary outcome will be a measure of utilization that is similar to what Haynes *et al*. (2006) used in their trial of the McMaster Premium Literature Updating Service (PLUS) [39]. Specifically, we will track utilization at the organizational level by calculating the mean number of logins/month/organization (the total organizational logins/month will be averaged across the number of users from each organization) across trial groups during each of the baseline period, intervention period, and crossover period. We will also provide related descriptive measures such as the mean number of logins/month for different types of positions within the organization (executive director, management and staff), the range of logins/month within the organization, the proportion of organizations with at least one user accessing the 'full serve' and 'self-serve' versions of SHARE each month, the frequency with which systematic review records and related resources are accessed (*e.g.*, URLs to abstracts, user-friendly summaries, and/or full-text), and the number of times the email updates to the 'full-serve' group are forwarded.

Each version of the evidence service will be hosted on the Ontario HIV Treatment Network server and for the duration of the trial will require a user login that will be used to link each participant's identification with their usage of the evidence service website and to their organization. SHARE is a new database that is not yet publicly available (it will be upon completion of the trial), which allows us to evaluate it without participants being able to gain access from a publicly available site. In addition, requiring a user login will help protect against contamination of the intervention and control group. However, we cannot protect fully against the possibility of participants from the organizations sharing information given that many may collaborate with each other.

**Figure 1:**
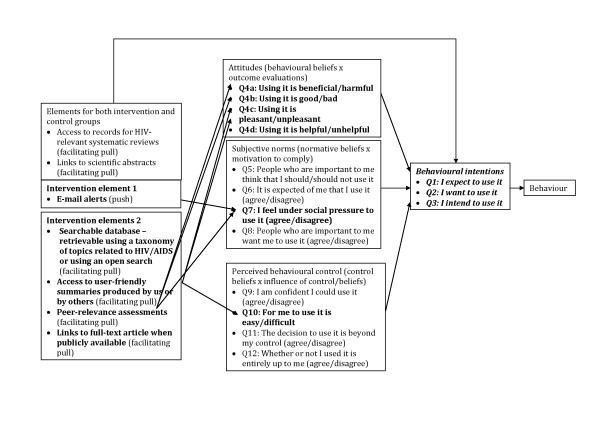
**Linkages among the intervention, contextual developments, and theory of planned behaviour constructs**.

For the secondary outcome, we will use the theory of planned behaviour to measure participants' intention to use research. The theory of planned behaviour proposes a model about how human action is guided [40,41] and consists of three variables -- attitudes (*i.e.*, beliefs and judgments), subjective norms (*i.e.*, normative beliefs and judgments about those beliefs), and perceived behavioural control (*i.e.*, the perceived ability to enact the behaviour) -- that shape the behaviour intentions of people, which is subsequently a strong predictor of future behaviour [41-43]. In Figure 1, we outline the model of the theory of planned behaviour and map how different elements of the evidence service may affect each of the three variables.

The theory of planned behaviour has been extensively used and tested in the fields of psychology and healthcare. Systematic reviews conducted in the psychology field have demonstrated that the theory explains about 39% of the variance in intention and about 27% of the variance in behaviour [42,43]. A number of studies have demonstrated the feasibility of producing valid and reliable measures of the key theory of planned behaviour constructs for use with healthcare professionals [44-46]. A systematic review suggests that the proportion of the variance in healthcare professionals' behaviour explained by intention was similar in magnitude to that found in the broader literature [47]. With the successful transfer of the theory from assessments of individuals to assessments of healthcare professionals involved in an agency relationship with their patients, we are confident in its further transfer to key decision makers in CBOs in agency relationships with other decision makers and staff in their organization.

Using a manual to support health researchers who want to construct measures based on the theory [41], our colleagues have developed and sought preliminary feedback on a data-collection instrument by first assessing face validity through interviews with key informants and then pilot testing it with 28 policymakers and researchers from 20 low- and middle-income countries who completed it after participating in a KT intervention [48]. In addition, Boyko *et al*. (2010) found moderate test-retest reliability of the instrument using Generalizability Theory (G = 0.50) [49] when scores from a sample of 37 health system policymakers, managers, professionals, citizens/consumers, and researchers participating in stakeholder dialogues convened by the McMaster Health Forum were generalized across a single administration, and even stronger reliability (G = 0.9) when scores were generalized across the average of two administrations of the tool [48]. In the reliability assessment by Boyko *et al*. (2010), the first administration of the tool immediately followed a McMaster Health Forum stakeholder dialogue, which may have promoted enthusiasm for using research evidence among participants. This likely produced higher measures of intention on the first administration of the tool as compared to the second, resulting in the lower G-score. Given that we won't be administering the tool in a similar atmosphere of enthusiasm for using research evidence, it is likely that the level of reliability of the tool will be sufficient without two administrations at both baseline and follow-up.

We have slightly modified the wording in each of the questions of the tool to reflect the different intervention being tested (SHARE) and the target audience (CBOs) (see Additional file 3: Appendix 3). We will administer the instrument to one key decision maker from each organization during the baseline period, as well as at the end of the six-month intervention period, through a brief online survey that takes approximately 10 minutes to complete. We will use unique identifiers for each participant to ensure their responses to the previous survey are linked for calculations of before-and-after changes in their intention to use research evidence. We will follow up with participants who do not complete the survey once per week for three weeks to minimize the number of participants lost to follow up.

### Data management and analysis

Data will be entered into SPSS 16.0 using unique identifiers that link each participant to their respective organizational identifier assigned during the randomization process. Analyses will be conducted by two members of the team (MGW and TB) and, during the analysis, all investigators -- except for one of us who is involved in the both the analysis and randomization (TB) -- will be blinded to the key linking the organizations to their unique identifiers.

We will treat both outcome measures as continuous variables and analyze the change in these measures over time using a two-way mixed effects linear repeated measures analysis of variance (ANOVA), which will assess the effects within groups, between groups, and over time with the latter as the main feature of interest. In addition, we will control for four variables -- province the organization is located in, size of organization (as measured by number full-time equivalent staff in the organization), number of participants that participated from each organization, and the number of clients served each year by the organization -- using analysis of covariance. For the analysis of the secondary outcome, we will also control for whether the key decision maker is full-time or part-time, and whether they have had research training in the past. Each of these variables may at least partially explain research use (*e.g.*, the amount of staff support an executive director or manager has may determine the extent to which they can spend time finding and using research evidence), and therefore adjusting for them will allow for a better assessment of the effects of the intervention. Moreover, as part of a secondary analysis, we will assess whether there is an interaction between each (entered as fixed factors) and the outcome measures. Given the likelihood that the distribution of the outcomes will be skewed, we will transform the data where necessary and possible, which may include adjusting the time period for which we calculate the mean number of logins/month/organization (*e.g.*, calculating the mean over two months) if the number of logins is low and there are insufficient data for analysis. We will also qualitatively compare the number of participants in the intervention and control groups that do not complete the follow-up survey, and attempt to assess if there are reasons for why they dropped out based on their baseline characteristics.

For all analyses, we will use the intention to treat principle, report 95% confidence intervals, and consider p-values equal to or less than 0.05 (two-tailed) to be significant. For the primary outcome measure (mean logins/month/organization), missing data are irrelevant as they are a naturalistic measure. For the secondary outcome measure (obtained through the survey), missing data can be taken into account through the use of a mixed-effects model.

### Statistical precision

Given a fixed sample size of approximately 85 organizations (70% of 120 organizations) a sample size calculation is not relevant. Instead, we have calculated the level of statistical precision that we can expect given our fixed sample size. To calculate the expected statistical precision in the trial, an estimation of intra-class correlation coefficient (ICC) of measurements for individuals over time for the primary outcome is required. However, we have no mechanism to estimate the ICC due to the fact that no similar study with this population has been conducted (at least to our knowledge). Therefore, we calculated estimates of statistical precision for ICCs of 0.2, 0.3, 0.5, 0.7 and 0.8 based on a six-month trial period with 80% power, an estimated standard deviation of 1.0, significance of 0.05 (two-sided test), and 42 organizations per study group (total n = 85) [50]. Assuming the primary outcome data will be collected from all 85 organizations during the intervention period at six follow-up points (one per month), the time-averaged detectible differences (in standard deviation units) between the two groups is at best 0.35 (for ICC = 0.2), which increases with successively greater ICCs to 0.39 (for ICC = 0.3), 0.47 (for ICC = 0.5), 0.53 (for ICC = 0.7), and 0.56 (for ICC = 0.8).

## Qualitative methods/design

Given that this is the first RCT evaluating a KTE intervention for CBOs (at least to our knowledge) and the inherent limitations associated with measuring research use as an outcome, we will conduct a qualitative process study after the completion of the trial to explore the RCT findings in greater depth. The qualitative study will explore how and why the evidence service worked (or didn't work), determine how the 'full-serve' and 'self-serve' evidence services were used, including the degree of contamination between the intervention and control groups, and other factors that may have influenced their use (*e.g.*, the ease of use of SHARE).

### Sample

We will use a mixed method sequential nested sampling procedure whereby a larger sample is analyzed in one study (RCT), and a subset of the larger sample is selected for further inquiry in the second study [51]. Specifically, one key organizational decision maker from 15 organizations in each trial arm (n = 30) will be purposively sampled [52,53]. First, we will divide the organizations according to whether they received the 'full-serve' or 'self-serve' evidence service. Next, we will purposively sample in order to obtain a breadth of perspective by ensuring there is a mix with different outcomes from the trial (*i.e.*, varying levels of research use and intention to use research), and with varying size and location within the country. We have assumed a 70% response rate, which means that we should sample approximately 40 organizational key decision makers to achieve a sample size of 30.

### Data collection

One-on-one semi-structured telephone interviews will be conducted with key decision makers about their experiences with the evidence service, including whether and how they used it (and the degree of 'contamination' between the two arms of the RCT, if any) and why, whether, and how it was helpful in their work and why, what aspects were most and least helpful and why, and recommendations for next steps. In addition, we will ask participants about any recommendations for how to improve upon our efforts to support the use of research evidence by CBOs. Finally, for the document analysis, we will collect all comments provided in the user forums for each systematic review record

### Data management and analysis

We will tape and transcribe all interviews, use N-Vivo 8 for data management of both the interview transcripts and document analysis, and use a constant comparative method for analysis [54-56]. Specifically, two reviewers will identify themes emerging from each successive wave of four to five interviews and iteratively refine the interview guide until we reach data saturation. This strategy will allow the reviewers to develop codes and broader themes in N-Vivo 8 that reflect the emerging and increasing levels of nuance that will inevitably result from the continuous checks that are involved in the constant comparative method [54,56]. We will also conduct member checking once analysis is completed (*i.e.*, we will send a brief, structured summary of what we learned from the interviews and invite comment on it). Finally, we will use the document analysis of the comments left in the user forum to help further our understanding of how participants engaged with the 'full-serve' version of SHARE.

## Discussion

To our knowledge, this will be the first RCT to evaluate the effects of an evidence service specifically designed to help CBOs find and use research evidence. As we have outlined elsewhere [21], efforts to support the use of research evidence by CBOs have been limited. In addition, rigorous evaluations of the effects of these strategies remains a critical gap in the KTE literature [21,24,57]. This study will begin to address this gap by providing a rigorous evaluation of the effects of a KTE intervention for CBOs, and by examining how and why the intervention succeeds or fails. In addition, this trial will complement a similar RCT we are planning to conduct with policy analysts and advisors in the Ontario Ministry of Health and Long-Term Care [58], and will contribute to an emerging evidence base about similarities and differences in 'what works' in KTE across different target audiences [13,14,59].

The main limitation of this trial is the relatively small sample size that we have available to draw upon. However, while the sample size is relatively small, we are still reaching an entire sector of CBOs, which will help provide more generalizable results. In addition, through our partnership with the Canadian AIDS Society and their support with study recruitment, we hope to achieve a high response rate. Another potential limitation is study contamination between the intervention and control groups as some participants may collaborate with each other and share their login and password. To assess contamination we have included a question in the follow-up survey asking if they shared their login and password with anyone else outside their organization.

## Competing interests

Three of the authors (MGW, JNL and SBR) were involved in the development of the SHARE database and remain involved in its continuous updating. SHARE is the intervention being tested in the trial.

## Authors' contributions

MGW conceived of the study, participated in its design, and drafted the protocol. JNL participated in the design of the study and helped draft the protocol. JG and RBH participated in the design of the study and provided feedback on drafts of the protocol. TB participated in the design of the study, performed the sample-size calculations, and provided feedback on drafts of the protocol. SBR provided feedback on drafts of the protocol. All authors read and approved the final manuscript.

## Supplementary Material

Additional file 1 **Appendix 1: SHARE (Synthesized HIV/AIDS Research Evidence) taxonomy of topics**. Topics used to categorize systematic reviews contained in SHARE Click here for file

Additional file 2 **Appendix 2: Peer-relevance assessment question**. Each systematic review record in SHARE asks users to answer one question about how useful the information is. The results are displayed to the user after answering the question. Click here for file

Additional file 3 **Appendix 3: Data collection instrument (secondary outcome measure)**. A survey measuring participants' intention to use research evidence, which will be administered at baseline and at the end of the trial. Click here for file
