# Supplementary material for: Effects of an evidence service on community-based AIDS service organizations' use of research evidence: A protocol for a randomized controlled trial
Source: Implement Sci. 2011 May 27;6:52. doi: 10.1186/1748-5908-6-52 (PMC3127774; doi:10.1186/1748-5908-6-52)
Supplement: Additional file 1 — Appendix 1: SHARE (Synthesized HIV/AIDS Research Evidence) taxonomy of topics. Topics used to categorize systematic reviews contained in SHARE [file 1748-5908-6-52-S1.DOC]

**Additional file 1**

**Appendix 1: SHARE (Synthesized HIV/AIDS Research Evidence) taxonomy of topics**

**Description**: Topics used to categorize systematic reviews contained in SHARE

## 1. People and context

## Jurisdiction(s) studied:

- High income country
- Low- and middle-income country
- Not reported

*Specify Country/continent*

- North America
- Canada
- United States
- Europe
- Africa
- Asia
- Australasia
- Latin America & Caribbean

## Population of interest

- General adult population
- Men who have sex with men
- Heterosexual men
- Women
- Pregnant women
- Aged/older adults
- Youth (15-24)
- Childhood (5-14 years)
- Infancy/Early Childhood (birth - 4 years)
- Aboriginal people
- Injection drug users
- Drug users (non-injecting)
- Ethnocultural communities
- Transgendered communities
- Immigrants
- Refugees
- Prisoners
- Sex workers
- Homeless/marginally housed communities
- Not specified

## Co-morbidities and infections

- - Mental health and addictions
    - Depression
    - Neurocognitive function
    - Severe and persistent mental illness
    - Personality disorders
    - Injection drug use/users
    - Other drug use/users
    - Alcohol
    - AIDS-related mental illness (e.g., dementia)
    - Co-infections
    - Hepatitis C
    - Hepatitis A or B
    - Sexually transmitted infections
    - Tuberculosis
    - Malaria
  - Treatment side effects
  - AIDS related lymphomas
  - HIV*/*HAART associated dyslipidemic lipodystrophy (HADL)
  - Other co-morbidities/co-occurring illnesses

## 2. Problem/issue definition

- Testing/Detection/Diagnosis
- Epidemiology (monitoring the burden and spread of HIV)

**3. Options for addressing a problem or issue**

## Determinants of health

- Income and Social Status
- Social Support Networks
- Education and Literacy
- Employment/Working Conditions
- Social Environments
- Physical Environments
- Personal Health Practices and Coping Skills
- Healthy Child Development
- Biology and Genetic Endowment
- Health Services
- Gender
- Culture

## Health system arrangements

- Governance arrangements
- Policy authority
- Organizational authority
- Commercial authority
- Professional authority
- Consumer & stakeholder involvement
- Financial arrangements
- Financing
- Funding
- Remuneration
- Financial incentives for patients
- Resource allocation
- Delivery arrangements
- To whom care is provided & with what efforts to reach them
- By whom care is delivered
- Where care is provided
- With what information & communication technology (ICT) is care provided
- With what quality & safety care is provided

## Programs, services & drugs within health systems

- Prevention
- Primary prevention
- Condoms
- Drug related (not harm reduction)
- Education/training
- Behavioural
- Mass media/campaigns
- Vaccines
- Circumcision
- Microbicides
- Pre-natal/Perinatal
- Pre & post-prophylaxis
- Secondary prevention
- Positive prevention
- Indirect (e.g., through identification or treatment of other STIs)
- Harm reduction
- Treatment & support
- Anti-retroviral
- Combination therapy (HAART)
- Single drug therapy
- Initiation of treatment
- Changing/stopping/interrupting treatment
- Salvage therapy
- Gene therapy
- Side effects
- Complementary therapy
- Nutrition
- Psychological
- Education
- Psychosocial/behavioural
- Opportunistic infections
- Exercise/rehabilitation

## 4. Implementation strategies

- Consumer-targeted strategy
- Information or education provision
- Behaviour change support
- Skills and competencies development
- (Personal) Support
- Communication and decision-making facilitation
- System participation
- Provider-targeted strategy
- Educational material
- Educational meeting
- Educational outreach visit
- Local opinion leader
- Local consensus process
- Peer review
- Audit and feedback
- Reminders and prompt
- Tailored intervention
- Patient-mediated intervention
- Multi-faceted intervention
- **Organization-targeted strategy**
