# Supplementary material for: Effects of an evidence service on community-based AIDS service organizations' use of research evidence: A protocol for a randomized controlled trial
Source: Implement Sci. 2011 May 27;6:52. doi: 10.1186/1748-5908-6-52 (PMC3127774; doi:10.1186/1748-5908-6-52)
Supplement: Additional file 2 — Appendix 2: Peer-relevance assessment question. Each systematic review record in SHARE asks users to answer one question about how useful the information is. The results are displayed to the user after answering the question. [file 1748-5908-6-52-S2.DOC]

**Additional file 2**

**Appendix 2: Peer-relevance assessment question**

**Description:** Each systematic review record in SHARE asks users to answer one question about how useful the information is. The results are displayed to the user after answering the question.

How useful is the information presented in this summary to decision-making in your organization or jurisdiction?

| Score | Criterion |
| --- | --- |
| - 5 | Highly useful: I would definitely use this |
| - 4 | Probably useful: I would likely use this |
| - 3 | Somewhat useful |
| - 2 | Probably not useful: I would likely not use this |
| - 1 | Not useful: I would definitely not use this |
