# Supplementary material for: Effects of an evidence service on community-based AIDS service organizations' use of research evidence: A protocol for a randomized controlled trial
Source: Implement Sci. 2011 May 27;6:52. doi: 10.1186/1748-5908-6-52 (PMC3127774; doi:10.1186/1748-5908-6-52)
Supplement: Additional file 3 — Appendix 3: Data collection instrument (secondary outcome measure). A survey measuring participants' intention to use research evidence, which will be administered at baseline and at the end of the trial. [file 1748-5908-6-52-S3.DOC]

**Additional file 3**

**Appendix 3: Data collection instrument (secondary outcome measure)**

**Description**: A survey measuring participants’ intention to use research evidence, which will be administered at baseline and at the end of the trial.

**Background**

We have developed an evidence service for community-based organizations working in the HIV sector entitled SHARE (Synthesized HIV/AIDS Research Evidence). SHARE provides access to records of syntheses of research evidence (systematic reviews, overviews of systematic reviews, and treatment guidelines) that address topics related to HIV/AIDS in order to help organizations more easily find and use research evidence in their work.

Before you access the evidence service, we would like you to complete a brief survey that should take you approximately 5 to 10 minutes to complete.

**Section A. Intention to use research evidence**

Each question in this section refers to a scenario where you need to make a decision about programs and services delivered by your organization, brief or provide advice to another manager in your organization or when you are involved in advocacy, a policy debate or deliberations with policymakers and/or other organizations. Please answer each question as though you are engaged in a typical decision-making process or advocacy initiative.

1. I expect to use synthesized research evidence of the type contained in SHARE to help work through what I will decide or advocate for.

| Strongly disagree | Disagree | Somewhat  disagree | Neither agree nor disagree | Somewhat  agree | Agree | Strongly agree |
| --- | --- | --- | --- | --- | --- | --- |
| 1 | 2 | 3 | 4 | 5 | 6 | 7 |

1. I want to use synthesized research evidence of the type contained in SHARE to help work through what I will decide or advocate for.

| Strongly disagree | Disagree | Somewhat  disagree | Neither agree nor disagree | Somewhat  agree | Agree | Strongly agree |
| --- | --- | --- | --- | --- | --- | --- |
| 1 | 2 | 3 | 4 | 5 | 6 | 7 |

1. I intend to use synthesized research evidence of the type contained in SHARE to help work through what I will decide or advocate for.

| Strongly disagree | Disagree | Somewhat  disagree | Neither agree nor disagree | Somewhat  agree | Agree | Strongly agree |
| --- | --- | --- | --- | --- | --- | --- |
| 1 | 2 | 3 | 4 | 5 | 6 | 7 |

1. Using synthesized research evidence of the type contained in SHARE to help work through what I will decide or advocate for is…

| Very harmful | Moderately  harmful | Slightly harmful | Neutral | Slightly beneficial | Moderately beneficial | Very beneficial |
| --- | --- | --- | --- | --- | --- | --- |
| 1 | 2 | 3 | 4 | 5 | 6 | 7 |

| Very bad | Moderately bad | Slightly bad | Neutral | Slightly good | Moderately good | Very good |
| --- | --- | --- | --- | --- | --- | --- |
| 1 | 2 | 3 | 4 | 5 | 6 | 7 |

| Very unpleasant  (for me) | Moderately  unpleasant (for me) | Slightly  unpleasant  (for me) | Neutral | Slightly pleasant (for me) | Moderately pleasant (for me) | Very pleasant (for me) |
| --- | --- | --- | --- | --- | --- | --- |
| 1 | 2 | 3 | 4 | 5 | 6 | 7 |

| Very unhelpful | Moderately  unhelpful | Slightly unhelpful | Neutral | Slightly helpful | Moderately helpful | Very helpful |
| --- | --- | --- | --- | --- | --- | --- |
| 1 | 2 | 3 | 4 | 5 | 6 | 7 |

1. Most people who are important to me in my professional life think that…

| I should definitely not | I should almost certainly not | I should probably not | Neutral | I should probably | I should almost certainly | I should definitely |
| --- | --- | --- | --- | --- | --- | --- |
| 1 | 2 | 3 | 4 | 5 | 6 | 7 |

…use synthesized research evidence of the type contained in SHARE to help work through what I will decide or advocate for..

1. It is expected of me that I use synthesized research of the type contained in SHARE evidence to help work through what I will decide or advocate for.

| Strongly disagree | Disagree | Somewhat  disagree | Neither agree nor disagree | Somewhat  agree | Agree | Strongly agree |
| --- | --- | --- | --- | --- | --- | --- |
| 1 | 2 | 3 | 4 | 5 | 6 | 7 |

1. I feel under social pressure to use synthesized research evidence of the type contained in SHARE to help work through what I will decide or advocate for.

| Strongly disagree | Disagree | Somewhat  disagree | Neither agree nor disagree | Somewhat  agree | Agree | Strongly agree |
| --- | --- | --- | --- | --- | --- | --- |
| 1 | 2 | 3 | 4 | 5 | 6 | 7 |

1. People who are important to me in my professional life want me to use synthesized research evidence of the type contained in SHARE to help work through what I will decide or advocate for.

| Strongly disagree | Disagree | Somewhat  disagree | Neither agree nor disagree | Somewhat  agree | Agree | Strongly agree |
| --- | --- | --- | --- | --- | --- | --- |
| 1 | 2 | 3 | 4 | 5 | 6 | 7 |

1. I am confident that I could use synthesized research evidence of the type contained in SHARE to help work through what I will decide or advocate for..

| Strongly disagree | Disagree | Somewhat  disagree | Neither agree nor disagree | Somewhat  agree | Agree | Strongly agree |
| --- | --- | --- | --- | --- | --- | --- |
| 1 | 2 | 3 | 4 | 5 | 6 | 7 |

1. For me to use synthesized research evidence of the type contained in SHARE to help work through what I will decide or advocate for is…

| Very difficult | Moderately difficult | Slightly difficult | Neutral | Slightly easy | Moderately easy | Very easy |
| --- | --- | --- | --- | --- | --- | --- |
| 1 | 2 | 3 | 4 | 5 | 6 | 7 |

1. The decision to use synthesized research evidence of the type contained in SHARE to help work through what I will decide or advocate for.

| Strongly disagree | Disagree | Somewhat  disagree | Neither agree nor disagree | Somewhat  agree | Agree | Strongly agree |
| --- | --- | --- | --- | --- | --- | --- |
| 1 | 2 | 3 | 4 | 5 | 6 | 7 |

1. Whether or not I use synthesized research evidence of the type contained in SAHRE to help work through what I will decide or advocate for.

| Strongly disagree | Disagree | Somewhat  disagree | Neither agree nor disagree | Somewhat  agree | Agree | Strongly agree |
| --- | --- | --- | --- | --- | --- | --- |
| 1 | 2 | 3 | 4 | 5 | 6 | 7 |

The question below refers to how useful you found the information from the evidence service. **[used only in the follow-up survey]**

1. I found the information from the evidence service to be useful.

| Strongly disagree | Disagree | Somewhat  disagree | Neither agree nor disagree | Somewhat  agree | Agree | Strongly agree |
| --- | --- | --- | --- | --- | --- | --- |
| 1 | 2 | 3 | 4 | 5 | 6 | 7 |

**Section B. Organization and participant characteristics [questions asked only at baseline or at follow-up noted in square brackets]**

1. What is the title of your position? (open ended) **[asked only on baseline survey]**
2. Are you currently in a full-time or part-time position? **[asked only on baseline survey]**
3. Do you have previous training in research? **[asked only on baseline survey]**

- Yes (if yes, please briefly describe – open ended box provided)
- No

1. Did you participate in the workshop on finding and using research evidence provided by the Ontario AIDS Network (OAN) and the Ontario HIV Treatment Network at the OAN meeting of board chairs and executive directors in November 2009? **[asked only on baseline survey]**

- Yes
- No

1. In what province/territory is your organization located? **[asked only on baseline survey]**
   - British Columbia
   - Alberta
   - Saskatchewan
   - Manitoba
   - Ontario
   - Quebec
   - New Brunswick
   - Nova Scotia
   - Prince Edward Island
   - Newfoundland & Labrador
   - Yukon
   - Northwest Territories
   - Nunavut
2. Where does your organization principally provide services (choose only one)? **[asked only on baseline survey]**
   - Locally (*i.e.*, within one city or town)
   - Regional area (*i.e.*, sub-provincial area)
   - Entire province/territory
   - National/multi-province/territory
3. What type of area(s) does your organization serve (please choose the one that best describes your area)? **[asked only on baseline survey]**
   - Urban
   - Rural
   - Both urban and rural
   - Northern and remote
4. Approximately how many paid full-time equivalent staff for HIV-related work are there in your organization? **[asked only on baseline survey]**

- 0 to 5
- 6 to 10
- 11 to 15
- 16 to 20
- 21 to 25
- 26 to 30
- 30+

1. Approximately how many people does your organization directly serve each year (*i.e.*, not just HIV related) **[asked only on baseline survey]**

- 0 to 250
- 251 to 500
- 501 to 750
- 751 to 1000
- 1001 to 1500
- 1501 to 2000
- 2000+

1. Did someone other than participant enrolled in the study complete this survey? **[asked only on follow-up survey]**

- Yes
- No

1. What is the title of the position of the person who filled out the survey (if you answered ‘Yes’ to question 10) **[asked only on follow-up survey]**
2. Were you also the person that filled out the original survey? If no, please what is the title of the person who filled out the original survey? **[asked only on follow-up survey]**

- Yes
- No (open-ended response provided for title of position)

1. Did you provide your login ID and password to anyone else outside of your organization during the study in order to support their use of research evidence? **[asked only on follow-up survey]**

- Yes (if yes, how many?)
- No

1. Did you provide your login ID and password to anyone else within your organization during the study in order to support their use of research evidence? **[asked only on follow-up survey]**

- Yes (if yes, how many?)
- No

1. Since the trial began, did you change part-time versus full-time status or position within your organization? If yes, please describe the change. **[asked only on follow-up survey] [open ended response]**

- Yes (if yes, please describe – space for open-ended response provided)
- No

1. Since the trial began, did you take any vacation or leave that lasted more than two weeks? If yes, please describe the duration and timing of the vacation or leave **[asked only on follow-up survey]**

- Yes (open-ended box provided for description of duration and timing of leave)
- No
